# Supplementary material for: Global inequity creates local insufficiency: A qualitative study of COVID-19 vaccine implementation challenges in low-and-middle-income countries
Source: PLoS One. 2023 Feb 13;18(2):e0281358. doi: 10.1371/journal.pone.0281358 (PMC9925066; doi:10.1371/journal.pone.0281358)
Supplement: Attachment — Submitted filename: Response to reviewers_Plos GPH _Delphi content analysis.docx [file pone.0281358.s003.docx]

**Response to Reviewer Comments**

**January 5, 2022**

Dear Dr Saurav Basu,

Thank you for the opportunity to submit a revision of our manuscript, entitled “Global inequity creates local insufficiency: A qualitative study of COVID-19 vaccine implementation challenges in low-and-middle-income countries” for consideration at *PLOS One*.

We thank you and the reviewers for their comments on this manuscript. We have made changes to the manuscript as per the suggestions, which are detailed below. We feel the paper is much improved because of these changes and hope that the reviewers, and you as editor, will agree with these revisions.

We hope these amendments have strengthened the manuscript and that it is suitable for publication at *PLOS One.*

Sincerely,

Ross Upshur

**Journal Requirements**:

**Comment 1.** Please ensure that your manuscript meets PLOS ONE's style requirements, including those for file naming. The PLOS ONE style templates can be found at

<https://journals.plos.org/plosone/s/file?id=wjVg/PLOSOne_formatting_sample_main_body.pdf>  and <https://journals.plos.org/plosone/s/file?id=ba62/PLOSOne_formatting_sample_title_authors_affiliations.pdf>

**Response 1.** We have updated our manuscript to adhere to PLOS ONE’s style requirements

**Comment 2**. In your Data Availability statement, you have not specified where the minimal data set underlying the results described in your manuscript can be found. PLOS defines a study's minimal data set as the underlying data used to reach the conclusions drawn in the manuscript and any additional data required to replicate the reported study findings in their entirety. All PLOS journals require that the minimal data set be made fully available. For more information about our data policy, please see <http://journals.plos.org/plosone/s/data-availability>.

Upon re-submitting your revised manuscript, please upload your study’s minimal underlying data set as either Supporting Information files or to a stable, public repository and include the relevant URLs, DOIs, or accession numbers within your revised cover letter. For a list of acceptable repositories, please see <http://journals.plos.org/plosone/s/data-availability#loc-recommended-repositories>. Any potentially identifying patient information must be fully anonymized.

Important: If there are ethical or legal restrictions to sharing your data publicly, please explain these restrictions in detail. Please see our guidelines for more information on what we consider unacceptable restrictions to publicly sharing data: <http://journals.plos.org/plosone/s/data-availability#loc-unacceptable-data-access-restrictions>. Note that it is not acceptable for the authors to be the sole named individuals responsible for ensuring data access.

We will update your Data Availability statement to reflect the information you provide in your cover letter.

**Response 2:** We have provided a de-identified version of the data set. It is available here:

Haldane, V; Ariyarajah, Archchun; Berry, Isha; Loutet, Miranda; SALAMANCA-BUENTELLO, FABIO; Upshur, Ross EG (2023): Round 1 Delphi responses (de-identified) - COVID-19 vaccine consensus study (LMICs). figshare. Dataset. <https://doi.org/10.6084/m9.figshare.21825135.v1>

**Comment 3.** Please review your reference list to ensure that it is complete and correct. If you have cited papers that have been retracted, please include the rationale for doing so in the manuscript text or remove these references and replace them with relevant current references. Any changes to the reference list should be mentioned in the rebuttal letter that accompanies your revised manuscript. If you need to cite a retracted article, indicate the article’s retracted status in the References list and also include a citation and full reference for the retraction notice.

**Response 3.** We have reviewed the reference list and all is in order.

**Comment 4.** Also, any publications (published or under review) associated with the same dataset need to be clarified with reasons.

**Response 4.** The complete results of the Delphi have previously been published as:

Ariyarajah A, Berry I, Haldane V, Loutet M, Salamanca-Buentello F, Upshur RE. Identifying priority challenges and solutions for COVID-19 vaccine delivery in low-and middle-income countries: A modified Delphi study. PLOS Global Public Health. 2022 Sep 8;2(9):e0000844. Available at: <https://journals.plos.org/globalpublichealth/article?id=10.1371/journal.pgph.0000844#references>

The manuscript currently under review is a companion to this Delphi. Whereas the full Delphi process leads to consensus, in the initial phase of the study many participant perspectives were collected that offer a breadth of important considerations for vaccine implementation in low- and middle-income settings. This study uses qualitative methods to explore these perspectives and in doing so differs significantly from the Delphi and sheds different light on COVID-19 vaccine program implementation.

**Reviewer #1:**

**Comment 5.** The data should be made fully available or explicitly justify not making the data available.

**Response 5.** TBD.

**Comment 6.** There are a few minor typos, e.g., page 4 that says, "Figure 1 about here" that should be corrected. The grammar is solid overall but would benefit from a final review.

**Response 6.** Thank you, we have copy edited the document. We have removed the placeholder text for figure 1.

**Comment 7.** The authors should explain why they have broken their data out into multiple publications, including only the Round 1 data here. It would help to have all of the rounds in context here or justify why the other rounds are not included.

**Response 7.** We have chosen to focus on our Round 1 data in this manuscript as it offers a breadth of perspectives on COVID-19 vaccine program implementation. The goal of the Delphi is to generate consensus, which while important, by nature distills and ranks many challenges and solutions into only a few. It is important that the diverse perspectives offered in the free text responses of the first phase have a place to be explored and discussed, which is not within the scope or aims of Delphi, but which is well suited to qualitative analysis as presented here. All rounds of data are not included here as only the first round involves gathering qualitative data – subsequent rounds use quantitative methods to determine agreement and ranking, thus making these data inappropriate for content analysis.

**Comment 8.** Please identify the countries represented in the study for context (even a range of how many countries are represented would be helpful).

**Response 8.** We have reported on the region of expertise of the panel that answered the survey questions in Table 1. We asked in this way as many participants in our target population have expertise across several countries, as well as challenges in the distinction between an expert’s country of origin, country of residence, or country where they work. Phrasing it as country of expertise and allowing for multiple selections allows our participants to self-identify.

**Comment 9.** Please list the response rate to invitations to participate in the study.

**Response 9.** Thank you for bringing this to our attention. We have now added the response rate in the results section, line 174. The section now reads “A total of 426 participants were invited to be involved in the Delphi study. Of these, 96 (22%) completed Round I of the survey and provided responses that were included in the content analysis.”

**Reviewer #2:**

**Comment 10.**  This manuscript is well written. I only have concerns for some 'over-looked' absurdities.

For instance, some sections of the tables that bear the results of the study still retain the flavour of the survey instrument, e.g. 'select all that apply'. You should read through the entire paper and eliminate typographical issues from it.

Overall, this is a very good paper on the subject matter. It has established that global inequity is a driver of COVID-19 vaccine programme implementation insufficiency in LMICs. I am sure that the paper, when published, will be a good addition to the efforts that are being directed at recovery from the pandemic.

**Response 10.** Thank you for your support of the manuscript. We have clarified in table 1 that the ‘select all that apply’ is an indication that participants could select as many regions as they felt were their region of expertise. The title of the category now says, “Region of expertise (participants could select multiple regions).” We hope this clarifies and adequately addresses the reviewer’s concern.
